# Complete Genome Sequence of a Recombinant NADC30-Like Strain, SCnj16, of Porcine Reproductive and Respiratory Syndrome Virus in Southwestern China

**DOI:** 10.1128/genomeA.00004-18

**Published:** 2018-02-08

**Authors:** Long Zhou, Runmin Kang, Bo Xie, Yiming Tian, Xin Yang, Jifeng Yu, Hongning Wang

**Affiliations:** a School of Life Science, Sichuan University, Animal Disease Prevention and Food Safety Key Laboratory of Sichuan Province, Key Laboratory of Bio-Resources and Eco-Environment, Ministry of Education, Chengdu, China; b Sichuan Animal Science Academy, Sichuan Provincial Key Laboratory of Animal Breeding and Genetics, Chengdu, China; c Chengdu Chia Tai Agro-Industry & Food Co., Ltd, Animal Healthy Disease Service, Chengdu, China

## Abstract

The NADC30-like strains of porcine reproductive and respiratory syndrome virus (PRRSV) are characterized by a 131-amino-acid deletion in nonstructural protein 2 (NSP2). Here, we report the complete genome sequence of a recombinant NADC30-like PRRSV strain, SCnj16, that exhibits the molecular marker of the Chinese highly pathogenic PRRSV (HP-PRRSV) in NSP2.

## GENOME ANNOUNCEMENT

Porcine reproductive and respiratory syndrome (PRRS) is an economically significant infectious disease in swine worldwide. The etiological agent, PRRS virus (PRRSV), is an enveloped, positive-sense RNA virus that is classified in the order *Nidovirales*, family *Arteriviridae*. PRRSV can be divided into two genotypes, the European genotype 1 and the North American genotype 2 with a divergence of approximately 60% at the nucleotide level (1–3). In 2006, a highly pathogenic PRRSV (HP-PRRSV) with a discontinuous 30-amino-acid (aa) deletion (1 aa and 29 aa) in nonstructural protein 2 (NSP2) emerged in China, which caused huge economic losses in the swine industry (4). In recent years, NADC30-like PRRSVs, which emerged in large areas of China, showed a discontinuous 131-aa deletion (111 aa, 1 aa, and 19 aa) in NSP2 relative to the sequence of strain VR-2332 (5, 6). Here, we report the complete genome sequence of an NADC30-like PRRSV strain, SCnj16, that exhibits the molecular marker in NSP2 of the Chinese HP-PRRSV.

The PRRSV strain SCnj16 was isolated in 2016 from lung samples from diseased pigs in southwestern China. The virus was inoculated into porcine alveolar macrophages (PAMs) for viral propagation as previously described (7). The complete genomic sequences of SCnj16 were amplified with 12 overlapping fragments by reverse transcription-PCR (RT-PCR). The amplified PCR fragments were purified and cloned into a pMD19-T vector (TaKaRa, China) and then sequenced three times by an automated sequencer (Genetic Analyzer 3730XL; Applied Biosystems). Sequences were assembled into the complete genomic sequence using DNAStar software (Lasergene). The 5′- and 3′-terminal segments were amplified using 5′ and 3′ full rapid amplification of cDNA ends (RACE) kit (TaKaRa), respectively. Sequence alignments were performed using ClustalW. A phylogenetic tree was constructed using the neighbor-joining method in MEGA 6.0. The recombination events were analyzed by the SimPlot program (version 3.5.1), with a window size of 200 bp and a step size of 20 bp.

The complete genomic sequence of SCnj16 is 15,321 nucleotides (nt) in length, excluding the poly(A) tail. The genetic analyses revealed that SCnj16 exhibits 89.6%, 87.9%, 85.7%, and 87.8% nucleotide identity with the PRRSV strains JXA1, CH-1a, VR-2332, and NADC30, respectively, and only 54.4% nucleotide identity with the European prototype, Lelystad virus (LV). Unlike other NADC30-like PRRSVs, SCnj16 displays the discontinuous 30-aa deletion in NSP2, but at the whole-genome level, this variant was designated an NADC30-like strain. Based on the results of the similarity plot and phylogenetic analysis, we speculate that SCnj16 likely originated from recombination events between the NADC30-like and Chinese HP-PRRSV strains at three recombination breakpoints; the first was located in *nsp2* (at 3,006 nt), the second in *nsp3* (at 5,286 nt), and the third in *nsp9* (at 9,146 nt). SCnj16 had different recombination patterns from the reported NADC30-like strains isolated in China from 2013 to 2016 (5, 6, 8, 9), indicating the emergence of new PRRSV recombination variants in the region. The genomic data of SCnj16 revealed that the *nsp2* gene continued to undergo evolution by mutation and/or recombination, and the 30-aa deletion in NSP2 is no longer a unique molecular marker for Chinese HP-PRRSV.

### Accession number(s).

The complete genome sequence of the SCnj16 strain is available in GenBank under the accession no. MF196906.
